# Affibody-based molecular probe ^99m^Tc-(HE)_3_Z_HER2:V2_ for non-invasive HER2 detection in ovarian and breast cancer xenografts

**DOI:** 10.1515/med-2024-1027

**Published:** 2024-09-03

**Authors:** Xianwen Hu, Hongyu Hu, Dandan Li, Pan Wang, Jiong Cai

**Affiliations:** Department of Nuclear Medicine, Affiliated Hospital of Zunyi Medical University, Huichuan, Zunyi, 563003, China; Department of Obstetrics, Zunyi Hospital of Traditional Chinese Medicine, Zunyi, Guizhou, Zunyi, 563003, China; Department of Nuclear Medicine, Affiliated Hospital of Zunyi Medical University, 149 Dalian Road, Huichuan, Zunyi, 563003, China

**Keywords:** human epidermal growth factor receptor 2, affibody, SPECT imaging, ovarian cancer, ^99m^Tc

## Abstract

**Purpose:**

This study aimed to assess the biodistribution and bioactivity of the affibody molecular probe ^99m^Tc-(HE)_3_Z_HER2:V2_, prepared by genetic recombination, and to investigate its potential for targeted human epidermal growth factor receptor 2 (HER2) imaging in SKOV3 ovarian cancer and MDA-MB-361 breast cancer xenografts.

**Methods:**

Affibody molecules were generated through genetic recombination. The radiochemical purity of the ^99m^Tc-labeled HER2 affibody was determined using reverse phase high performance liquid chromatography (RP-HPLC). Evaluation of HER2 affinity in SKOV3 ovarian cancer cells and MDA-MB-361 breast cancer cells (HER2-positive) was conducted by calculating equilibrium dissociation constants. Biodistribution of the ^99m^Tc-labeled affibody molecular probe was assessed in Balb/c mice bearing SKOV3 tumors. Tumor targeting specificity was evaluated in Balb/c mice using SKOV3, MDA-MB-361, and AT-3 (HER2-negative) xenografts.

**Results:**

Affibody (HE)_3_Z_HER2:V2_, generated through recombinant gene expression, was successfully labeled with ^99m^Tc, achieving a radiochemical purity of (96.0 ± 1.7)% (*n* = 3) as determined by RP-HPLC. This molecular probe exhibited specific binding to HER2-positive SKOV3 cells, demonstrating intense radioactive uptake. Biodistribution analysis showed rapid accumulation of ^99m^Tc-(HE)_3_Z_HER2:V2_ in HER2-positive tumors post-administration, primarily clearing through the urinary system. Single-photon emission computed tomography imaging conducted 1–3 h after intravenous injection of ^99m^Tc-(HE)_3_Z_HER2:V2_ into HER2-positive SKOV3 and MDA-MB-361 nude mouse models confirmed targeted uptake of the molecular probe by the tumors.

**Conclusions:**

The molecular probe ^99m^Tc-(HE)_3_Z_HER2:V2_ developed in this study effectively targets HER2 for imaging HER2-positive SKOV3 and MDA-MB-361 xenografts *in vivo*. It exhibits rapid blood clearance without evident toxic effects, suggesting its potential as a valuable marker for detecting HER2 expression in tumor cells.

## 1 Introduction

Human epidermal growth factor receptor 2 (HER2) is a transmembrane tyrosine kinase protein belonging to the epidermal growth factor receptor family. It consists of three main domains: the extracellular domain (ECD), a single-chain transmembrane region, and an intracellular protein tyrosine kinase region. Unlike other members of its family, HER2 does not have identified ligands that directly bind to it; instead, its activation occurs primarily through heterodimerization with other family members or via proteolytic cleavage of ECD proteins [1]. HER2 gene amplification or overexpression is observed in various human cancers, including breast, ovarian, fallopian tube, endometrial, and gastric cancers. This genetic alteration leads to accelerated cancer cell proliferation, enhanced DNA repair capabilities, and rapid tumor progression, significantly impacting patient prognosis [2,3]. Consequently, HER2 is a crucial therapeutic target in oncology, necessitating accurate detection of HER2-positive expression for precise diagnosis and targeted therapy. Currently, clinical assessment of HER2 receptor status often involves invasive procedures such as biopsy of the lesion followed by immunohistochemistry analysis or fluorescence *in situ* hybridization [4]. However, these methods are limited in their ability to provide real-time detection of systemic lesions and monitoring of HER2 levels due to the heterogeneous expression of HER2 within tumors, which can change with disease progression [5]. Furthermore, frequent needle biopsies are impractical in clinical settings due to patient discomfort and intolerance to repeated procedures.

In molecular-targeted nuclear medicine imaging, such as single-photon emission computed tomography (SPECT) and positron emission tomography (PET), imaging is conducted at the cellular and molecular levels using radionuclide-labeled imaging agents or therapeutic drugs designed to target specific molecules within tumor cells or gene fragments. These agents are typically administered via intravenous injection, allowing them to reach and bind specifically to their intended targets for imaging or therapeutic purposes. PET imaging offers high spatial resolution, excellent sensitivity, and quantification capabilities, making it clinically valuable for early diagnosis, tumor staging and restaging, treatment guidance, assessment of treatment response, and prediction of tumor recurrence [6]. In contrast, SPECT utilizes single photon-emitting radioactive nuclides as imaging agents. Despite lower spatial resolution compared to PET, SPECT is more widely accessible and cost-effective. SPECT tracers do not require production using accelerators and often have longer half-lives, facilitating their extensive use in clinical and basic research settings. Commonly used molecular imaging probes targeting HER2 include antibodies, small molecular polypeptides, and proteins [7]. Affibodies, a class of scaffold proteins with a molecular weight of approximately 6.5 kDa, are widely utilized as molecular recognition tools in both diagnostic and therapeutic applications [8–12]. Technetium-99m (^99m^Tc) is a cost-effective isotope obtainable from a ^99m^Mo–^99m^Tc generator by elution with normal saline. Affibodies typically feature glycine and cysteine residues that facilitate chelation of the ^99m^Tc nuclide. This study aimed to evaluate the biodistribution and targeting efficacy of ^99m^Tc-(HE)_3_Z_HER2:V2_ in Balb/c mice bearing SKOV3 and MDA-MB-361 tumors.

## 2 Methods

### 2.1 Production, radiolabeling of (HE)_3_Z_HER2:V2_

All chemicals used were commercially sourced. A novel HER2 affibody, (HE)_3_Z_HER2:V2_, was designed by appending HEHEHE to the amino terminus of the HER2 affibody Z_HER2:V2_ and synthesized via recombinant gene expression. Production and radiolabeling protocols for (HE)_3_Z_HER2:V2_ followed previously published methods [13]. The radiochemical purity of ^99m^Tc-(HE)_3_Z_HER2:V2_ was assessed using reversed-phase high-performance liquid chromatography (RP-HPLC, Shimadzu Instrument Co., Ltd, Suzhou, China) under the following conditions: Column: Agilent Eclipse Plus C18, 5 µm, 250 × 4.6 mm LC Column; column temperature: room temperature; flow rate: 1 mL/min. Solvent A consisted of acetonitrile with 0.1% trifluoroacetic acid, and solvent B comprised ultrapure water with 0.1% trifluoroacetic acid. A gradient elution program was employed: 10–60% A over 15 min.

### 2.2 *In vitro* stability of ^99m^Tc-(HE)_3_Z_HER2:V2_

^99m^Tc-(HE)_3_Z_HER2:V2_ (29.6 MBq/kg) was mixed with normal saline, PBS, and human serum at a ratio of 1:9, respectively, and incubated in a warm bath at 37°C. The radiochemical purity of ^99m^Tc-(HE)_3_Z_HER2:V2_ was assessed by TLC using 0.25 μL of each mixture at 0, 15, 30, 60, 120, 240, and 480 min. Instant thin-layer chromatography silica gel (iTLC-SG) chromatography paper from Agilent Technologies (Palo Alto, CA, USA) was employed.

### 2.3 Cell culture

The SKOV3 and MDA-MB-361 cell lines, characterized by high HER2 expression, were procured from Chongqing Boai Madison Biological Cell Center (Chongqing, China). These cells were cultured in RPMI 1640 Medium (from Beijing Biotechnology Co., Ltd, Beijing, China) supplemented with 1% penicillin–streptomycin and 10% fetal bovine serum (FBS; from Beijing Biotechnology Co., Ltd, Beijing, China) at 37°C in a 5% CO_2_ atmosphere. AT-3 cells, which do not express the HER2 receptor, were obtained from Shanghai Qingqi Biotechnology Development Co., Ltd (Shanghai, China) and cultured in DMEM medium supplemented with 10% FBS under similar conditions (37°C, 5% CO_2_).

### 2.4 Cellular uptake

We evaluated the cellular uptake and internalization of ^99m^Tc-(HE)_3_Z_HER2:V2_ using HER2-overexpressing ovarian cancer SKOV3 cells following methods outlined in our previously published literature [14]. Specifically, cells were seeded at a density of 10^6^ cells per well and incubated with labeled conjugates (1.5 nM) at 37°C. At predetermined time points (1, 2, 4, 8, 16, and 24 h post-incubation, *n* = 3), supernatants were collected from three wells, followed by two-fold washing of cells with ice-cold PBS to remove unbound radioligand. Combined fractions represented the unbound radioactivity. Cells were then placed on ice and treated with a buffer solution (pH 2.5) containing 4 M urea and 0.2 M glycine for 5 min to collect membrane-bound radioconjugates. Internalized affibodies were obtained after cell lysis with 1 M NaOH. The percentage of membrane-bound and internalized radioactivity was calculated for each time point. Non-specific binding was assessed by adding a 50-fold excess of unlabeled HER2 affibody under the same conditions. To confirm specific binding, the HER2-negative cell line AT-3 was used as a control, employing the identical experimental protocol as used for SKOV3 cells.

### 2.5 Saturation binding assay

Furthermore, different concentrations (1,658, 552.7, 184.2, 61.4, 20.5, 6.82, 2.27, and 0.76 nM, *n* = 3) of ^99m^Tc-(HE)_3_Z_HER2:V2_ were added to SKOV3 cells to determine the dissociation constant (*K*_d_). Cells were seeded in 24-well plates at a density of 4.6 × 10^5^ cells/mL with 1 mL/well of culture medium 24 h prior to the assay. On the following day, the cells were treated with the labeled ^99m^Tc-(HE)_3_Z_HER2:V2_, and each concentration was added in triplicate wells at a 1:2 dilution ratio. Additional 24-well plates treated under the same conditions used a 50-fold excess of unlabeled HER2 affibody as a blocking agent to assess non-specific binding. After incubation at 4°C for 1 h and washing with ice-cold PBS, the medium was removed, and cells were lysed with 0.4% NaOH. Cell lysates were collected and radioactivity measured using a gamma counter. Specific binding radioactivity counts were determined by subtracting counts from the blocking group from those of the non-blocking group. For MDA-MB-361 cells, different concentrations (33, 6.7, 1.31, 0.26, 0.054, 0.01, 0.0021, and 0.0004 nM) of ^99m^Tc-(HE)_3_Z_HER2:V2_ were similarly applied to calculate the *K*_d_ value, following the identical experimental protocol as for SKOV3 cells. The dissociation constant (*K*_d_) values were determined using GraphPad Prism 5 software, fitting the data to the equation *Y* = *B*_max_ × *X*/(*K*_d_ + *X*), where *X* represents the radioligand concentration, *Y* denotes specific binding radioactivity counts, and *B*_max_ signifies maximal binding radioactivity counts [15].

### 2.6 Animal model

All animal experiments were conducted in compliance with the Ethics Committee of the Affiliated Hospital of Zunyi Medical University (Approval Number: KLLY(A)-2020-074). Female Balb/C nude mice (6-week-old, weighing 18–20 g) were procured from Ensville Co., Ltd, Chongqing, China. The mice were housed in ventilated filter-topped cages with *ad libitum* access to standard diet and water throughout the experiment. Mice were randomly divided into three groups and implanted with approximately 1 × 10^6^ SKOV3, MDA-MB-361, or AT-3 tumor cells (in 100 μL of PBS) into the right axilla. The animals’ growth was monitored every 2 days, and tumor dimensions were measured using vernier calipers to calculate tumor volume using the formula: tumor volume = (*L* × *D* ^2^) × 1/2, where *L* is the longest diameter and *D* is the shortest diameter of the tumor. For biodistribution studies, SKOV3- and MDA-MB-361-bearing mice were used once tumor volumes reached approximately 1 cm^3^, typically 4–6 weeks post-injection.

### 2.7 *Ex vivo* biodistribution

The specific activity of ^99m^Tc-(HE)_3_Z_HER2:V2_ injected into the animals was 2.96 ± 0.24 GBq/μmol. The biodistribution of ^99m^Tc-(HE)_3_Z_HER2:V2_ and ^99m^Tc-ZHER2 in normal mice has been previously described [13]. For the biodistribution study in SKOV3 xenografts, mice received an injection of radioactive ^99m^Tc-(HE)_3_Z_HER2:V2_ at a concentration of 30.0 MBq/kg in a volume of approximately 40–60 μL via the tail vein. Subsequently, mice were sacrificed and dissected at 10, 30, 60, 120, and 240 min post-injection (5 mice per time point, totaling 25 mice). Tissue samples including blood, heart, liver, spleen, lung, kidney, stomach, intestine, bone, muscle, brain, thyroid, and tumor were collected and weighed. Radioactivity in each sample was measured using a γ-counter (Zhongjia Co., Ltd, Hefei, China), which features ten probes and ten channels capable of simultaneous measurement of ten sample tubes. Results were expressed as percentage of the injected dose per gram of tissue (% ID/g). For the SKOV3-blocked group, ^99m^Tc-(HE)_3_Z_HER2:V2_ with identical radioactive activity and 50 times the molar amount of unlabeled affibody (HE)3ZHER2 was administered via the tail vein. These animals were sacrificed and dissected 2 h post-injection. To minimize animal suffering, humane euthanasia was performed promptly via CO_2_ inhalation. Following euthanasia, animals were placed in lead-lined containers for 3 days and subsequently cremated after surface contamination measurements confirmed absence of residual radioactivity.

### 2.8 Scintigraphy and immunohistochemistry analyses

Balb/c nude mice bearing SKOV3- and MDA-MB-361-derived tumors (HER2-positive) or AT-3 tumors (HER2-negative) were included in the study (*n* = 3 per group). They received intravenous injections of ^99m^Tc-(HE)_3_Z_HER2:V2_ (40–60 µL, 30.0 MBq/kg), and SPECT imaging sessions were conducted at 1 and 3 h post-injection. In the SKOV3-blocked group, simultaneous injections were administered via the tail vein, consisting of equal radioactivity of ^99m^Tc-(HE)_3_Z_HER2:V2_ and a 50-fold excess of unlabeled affibody (HE)3ZHER2. Additionally, a control group received free ^99m^Tcvia tail vein injection into SKOV3-bearing mice, followed by imaging at 1 and 3 h post-injection. Following imaging, mice were euthanized using the aforementioned method, and tumor tissue samples were collected for immunohistochemical examination to confirm HER2 receptor expression in SKOV3, MDA-MB-361, and AT-3 xenografts. Scintigraphy was performed using a SPECT system (GE Healthcare, Chicago, IL, USA) with an acquisition time of 30 min and the following parameters: matrix size 256 × 256, zoom 4.0. Subsequent image analysis and processing were conducted using the GE Healthcare Xeleris™ Workstation.

### 2.9 Statistical analysis

All statistical analyses were conducted using SPSS software version 18.0. Measurement data, which were normally distributed, are presented as mean ± standard deviation (*x̄* ± s). For comparisons between two groups, independent samples *t*-tests were utilized, while one-way ANOVA was employed for comparisons involving multiple groups. Pairwise comparisons at *p* < 0.05 significance level were performed using the pairwise mean difference test (LSD method). In cases where variances were unequal, Tamhane’s T2 or Dunnett’s T3 tests were applied. A significance level of *p* < 0.05 was considered statistically significant.


**Ethical approval:** The study received approval from the ethics committee of the Affiliated Hospital of Zunyi Medical University (Zunyi, China) (grant number: KLLY(A)-2020-074), and all methods were conducted in accordance with ARRIVE guidelines.

## 3 Results

### 3.1 Radiochemical purity and stability of ^99m^Tc-(HE)_3_Z_HER2:V2_

**Figure 1:**
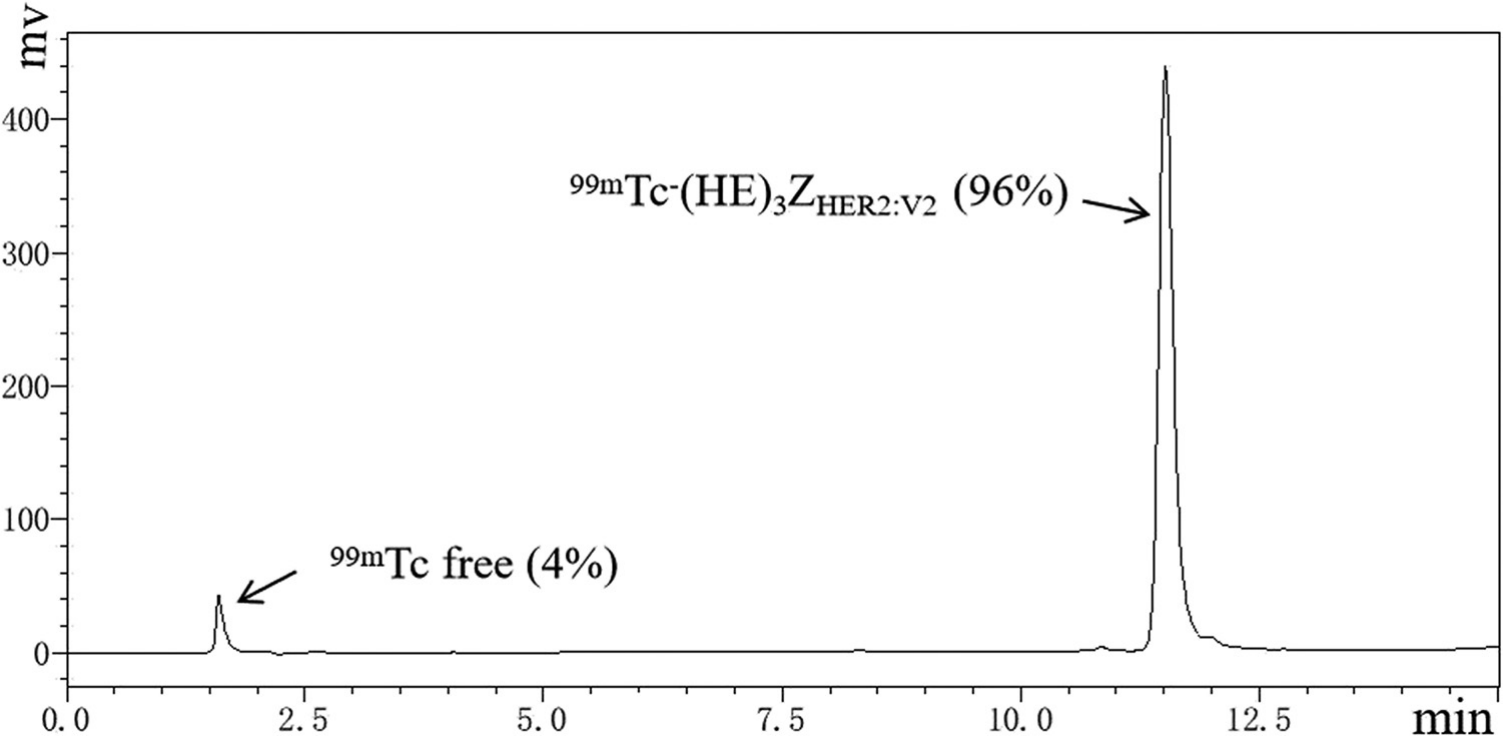
RP-HPLC chromatogram of ^99m^Tc-(HE)_3_Z_HER2:V2_. The radiochemical purity (the retention time of ^99m^Tc-(HE)_3_Z_HER2:V2_ = 11.5 min, and the retention time of the free ^99m^Tc was obtained at 1.5 min) was 96%.

**Figure A1:**
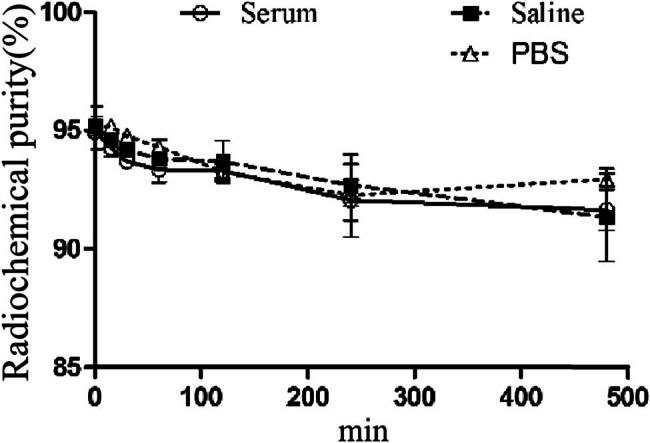
Stability of ^99m^Tc-(HE)_3_Z_HER2:V2_ in human serum, PBS and normal saline (*n* = 3); The radiochemical purity of ^99m^Tc-(HE)_3_Z_HER2:V2_ was determined by thin layer chromatography (TLC).

Affibody (HE)3ZHER2 was generated via recombinant gene expression and successfully labeled with ^99m^Tc, achieving a labeling yield of (97.2 ± 2.6)% (*n* = 5). RP-HPLC analysis of ^99m^Tc-(HE)_3_Z_HER2:V2_ indicated a radiochemical purity of (96.0 ± 1.7)% (*n* = 3), with a retention time of 11.5 min and a distinct peak. Free ^99m^Tc, used as a control, showed a retention time of 1.5 min (Figure 1). Stability studies demonstrated that ^99m^Tc-(HE)_3_Z_HER2:V2_ remained stable under various conditions, including normal saline, PBS, and human serum, after incubation at 37°C for 2 h. The radiochemical purity was maintained at levels exceeding 90% for up to 8 h (Figure A1). These findings confirm the *in vitro* stability of the labeled affibody.

### 3.2 Cellular uptake and binding specificity

**Figure 2:**
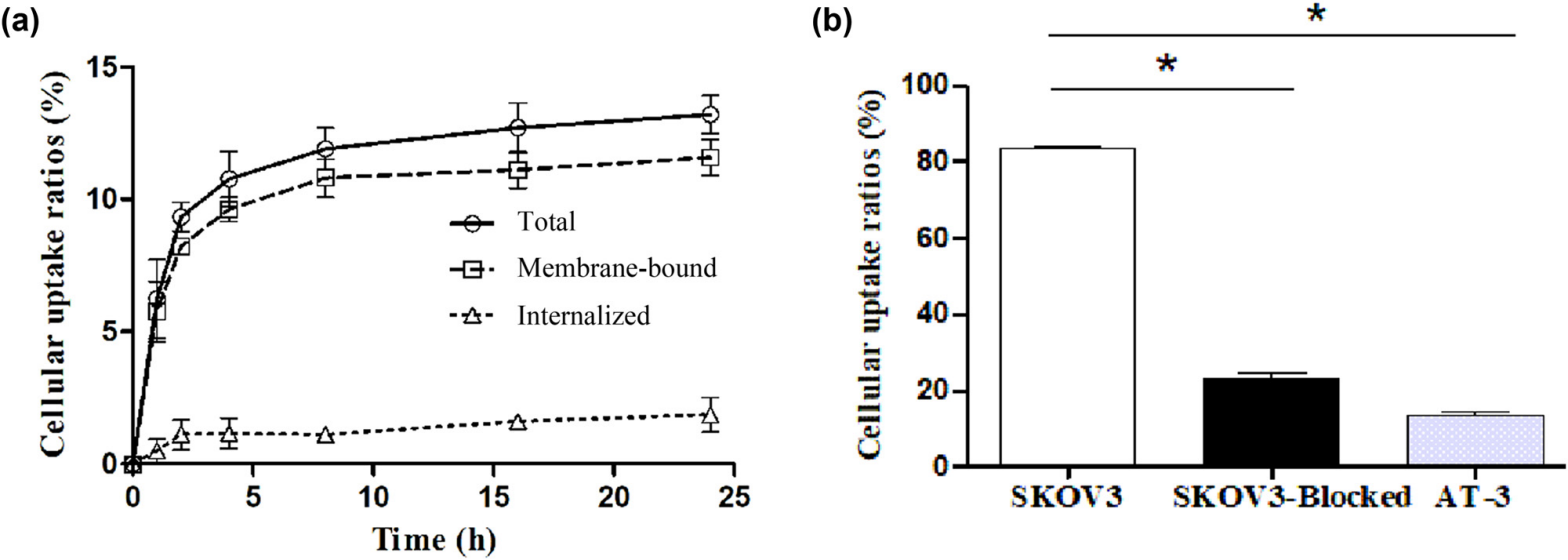
(a) Uptake and internalization of ^99m^Tc-(HE)_3_Z_HER2:V2_ at 37°C by SKOV3 cells (*n* = 3); (b) *In vitro* specificity analysis, specificity of ^99m^Tc-(HE)_3_Z_HER2:V2_ to SKOV3 cells (*n* = 3). Cell cultures were incubated with ^99m^Tc-Z_HER2:V2_ for 4 h. Blocked group of culture dishes with SKOV-3 cells was pretreated with saturating amounts of nonlabeled affibody before incubation with ^99m^Tc-(HE)_3_Z_HER2:V2_.The control group was AT-3 cells, *p < 0.05.

Binding of ^99m^Tc-(HE)_3_Z_HER2:V 2_ to SKOV3 cells increased rapidly during the first hour of culture and continued to rise gradually over time, reaching a plateau after 10 h. The internalization of ^99m^Tc-(HE)_3_Z_HER2:V2_ in SKOV3 cells remained below 2% at all observed times (Figure 2a). At all concentrations tested, the radioactivity in SKOV3 cells was significantly higher than in AT-3 cells at equivalent concentrations (*p* < 0.01). Competitive binding assays demonstrated that excess unlabeled affibody markedly reduced the binding of ^99m^Tc-(HE)_3_Z_HER2:V2_ to SKOV3 cells (Figure 2b), indicating receptor-mediated binding of ^99m^Tc-(HE)_3_Z_HER2:V2_ to living HER2-expressing cells.

**Figure 3:**
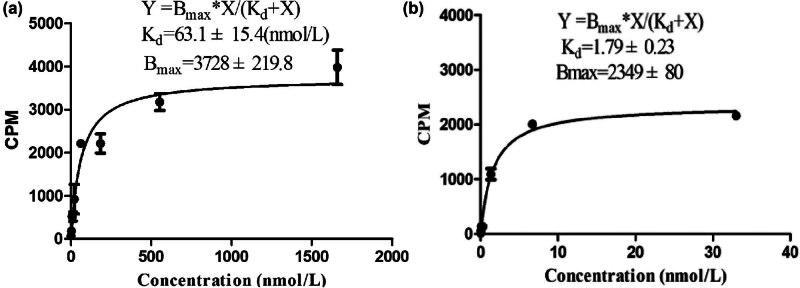
Assay of ^99m^Tc-(HE)_3_Z_HER2:V2_ binding affinity to SKOV3 (a) and MDA-MB-361 (b) cells (*n* = 3).

The saturation binding curve for *K*_d_ determination is shown in Figure 3. The *K*_d_ values of ^99m^Tc-(HE)_3_Z_HER2:V2_ binding to HER2 in SKOV3 and MDA-MB-361 cells were 63.1 ± 15.4 and 1.79 ± 0.23 nmol/L, respectively, demonstrating high-affinity specific binding of the radiolabeled molecular probe to HER2.

### 3.3 Biodistribution

The radioactivity uptake in HER2-positive SKOV3 xenografts at 10, 30, 60, 120, and 240 min was 6.44 ± 2.04% ID/g, 8.72 ± 1.58% ID/g, 9.71 ± 3.21% ID/g, 24.11 ± 7.90% ID/g, and 27.97 ± 7.73% ID/g, respectively (Table 1). Compared to the SKOV3 experimental group, the radioactive uptake in tumor tissue was significantly inhibited in the SKOV3 blocking group, consistent with results from *in vitro* uptake experiments. Blood concentration of ^99m^Tc-(HE)_3_Z_HER2:V2_ decreased rapidly from 2.42 ± 0.73% ID/g at 10 min to 0.45 ± 0.11% ID/g at 60 min. Furthermore, radioactive uptake in heart, liver, lung, spleen, bone, muscle, and other organ tissues also declined rapidly over time, consistent with previous biological distribution experiments in normal mice [13], resulting in a high tumor-to-background ratio. In addition to tumor radioactivity uptake, kidney uptake was also notably high, indicating primary excretion through the urinary system.

**Table 1:** Biodistribution of ^99m^Tc-(HE)_3_Z_HER2:V2_ in y in mice-bearing SKOV3 xenografts

Tissues (% ID/g)	SKOV3-^99m^Tc-(HE)_3_Z_HER2:V2_					SKOV3-blocked	*p* (120 min)
	10 min (*n* = 5)	30 min (*n* = 5)	60 min (*n* = 5)	120 min (*n* = 5)	240 min (*n* = 5)	120 min (*n* = 5)	
Heart	0.94 ± 0.31	0.38 ± 0.12	0.37 ± 0.06	0.31 ± 0.10	0.17 ± 0.05	0.21 ± 0.05	0.5682
Liver	12.48 ± 3.57	1.61 ± 0.42	1.50 ± 0.34	1.39 ± 0.32	1.33 ± 0.43	1.37 ± 0.26	0.8180
Spleen	2.34 ± 0.35	0.21 ± 0.04	0.22 ± 0.04	0.25 ± 0.12	0.20 ± 0.04	0.24 ± 0.01	0.7437
Lung	1.65 ± 0.51	1.19 ± 0.30	0.55 ± 0.13	0.34 ± 0.11	0.32 ± 0.06	0.40 ± 0.12	0.3586
Kidney	45.06 ± 13.61	26.81 ± 8.20	17.94 ± 5.34	16.75 ± 5.08	14.69 ± 3.90	14.11 ± 1.70	0.6552
Brain	0.14 ± 0.02	0.13 ± 0.04	0.10 ± 0.02	0.10 ± 0.04	0.08 ± 0.02	0.08 ± 0.01	0.2978
Muscle	4.27 ± 1.33	4.05 ± 1.06	1.60 ± 0.25	1.30 ± 0.35	1.11 ± 0.31	0.99 ± 0.32	0.4775
Bone	1.70 ± 0.40	1.24 ± 0.41	0.55 ± 0.18	0.45 ± 0.12	0.45 ± 0.13	0.62 ± 0.07	0.5683
Stomach	2.70 ± 0.86	1.58 ± 0.50	1.10 ± 0.34	1.09 ± 0.31	0.50 ± 0.05	0.90 ± 0.01	0.1756
Duodenum	0.54 ± 0.13	0.44 ± 0.11	0.37 ± 0.12	0.26 ± 0.08	0.22 ± 0.01	0.39 ± 0.11	0.4976
Thyroid	0.98 ± 0.17	0.54 ± 0.12	0.48 ± 0.12	0.44 ± 0.10	0.40 ± 0.05	0.48 ± 0.05	0.3688
Tumor	6.44 ± 2.04	8.72 ± 1.58	9.71 ± 3.21	24.11 ± 7.90	27.97 ± 7.73	4.07 ± 1.23	**<0.001**
Blood	2.42 ± 0.73	0.90 ± 0.23	0.45 ± 0.11	0.31 ± 0.10	0.24 ± 0.07	0.22 ± 0.06	0.2575

Note: % ID/g: percent injected dose rate per gram tissue.

Bold value indicate statistical significance.

**Figure 4:**
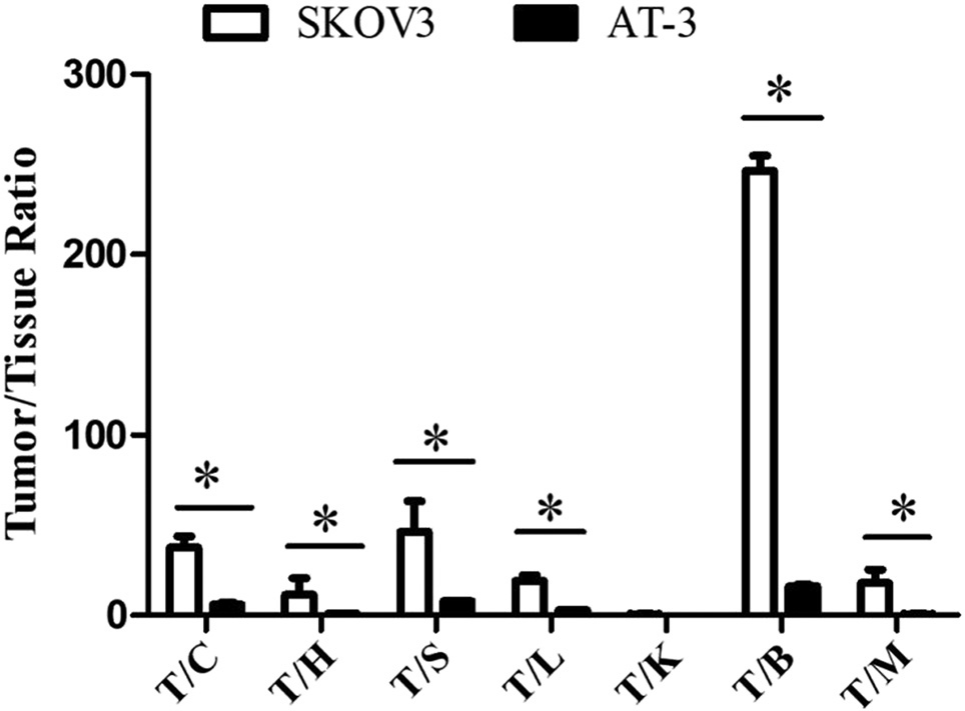
Tumor-to-tissue (target/non-target) ratio of ^99m^Tc-(HE)_3_Z_HER2:V2_ uptake in HER2-positive SKOV3 and HER2-negative AT-3 xenografts at 2 h after the injection of ^99m^Tc-(HE)_3_Z_HER2:V2_; *p < 0.05 (*n* = 5). Notes: T = tumor; C = cardiac; S = spleen; L = lung; K = kidney; B = brain; M = muscle.

The radioactive uptake ratio of HER2-positive SKOV3 tumor tissue to heart, liver, spleen, lung, brain, and muscle was significantly higher than that of HER2-negative AT-3 breast cancer (Figure 4), indicating specific binding of ^99m^Tc-(HE)_3_Z_HER2:V2_ to HER2.

### 3.4 Scintigraphy and immunohistochemistry

**Figure 5:**
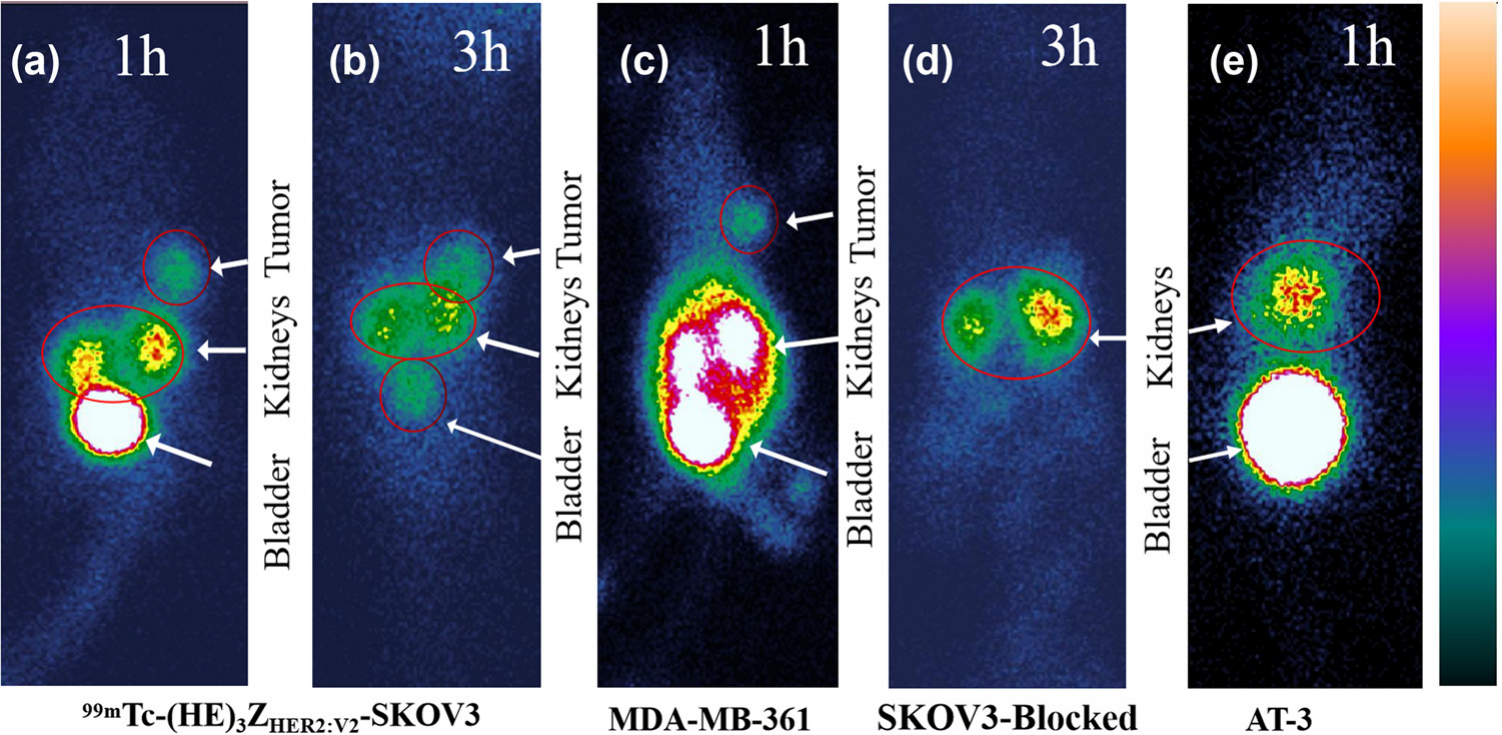
Imaging of HER2-positive expression in SKOV3 (a) and (b), MDA-MB-361 (c), SKOV3-blocked (d), and HER2-negative AT-3 (e) xenografts after the injection of ^99m^Tc-(HE)_3_Z_HER2:V2._

**Figure A2:**
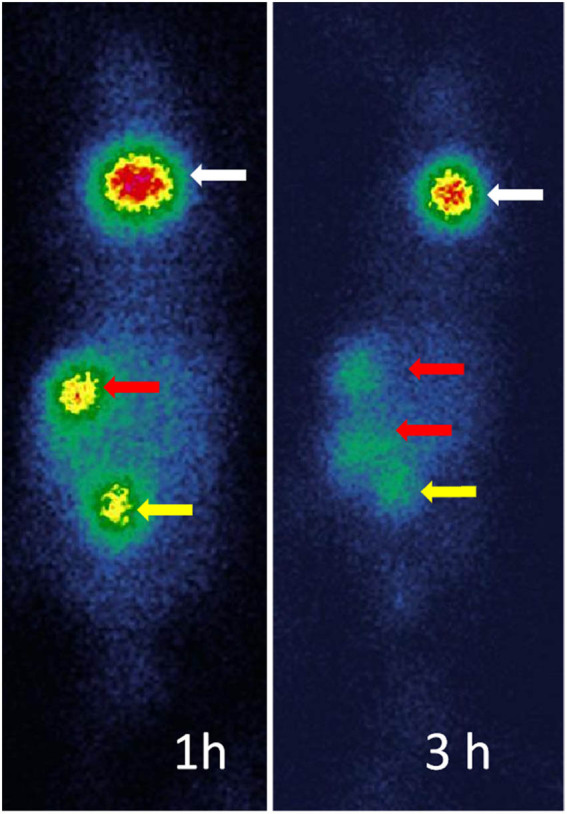
In the free ^99m^Tc control group, significant radiation uptake was observed in the thyroid (white arrows), kidney (red arrows), and bladder (yellow arrows), but no radiation uptake was observed in the tumor 1 and 3 h after injection of free ^99m^Tc.

Scintigraphy was conducted at 1 and 3 h post-injection of ^99m^Tc-(HE)_3_Z_HER2:V2_ in mice bearing SKOV3, MDA-MB-361, and AT-3 tumors, processed using Procreate (Apple, USA) software. Significant radioactive uptake was observed in HER2-positive SKOV3 tumors 1 h after injection, persisting at 3 h post-injection (Figure 5a and b). Similarly, substantial radioactive uptake was noted in HER2-positive MDA-MB-361 tumors at 1 h post-injection (Figure 5c). However, simultaneous injection of a 50-fold excess of unlabeled HER2 affibody blocked radioactive uptake in SKOV3 tumors at 3 h post-injection (Figure 5d). HER2-negative AT-3 tumors showed no radioactive uptake at any time following ^99m^Tc-(HE)_3_Z_HER2:V2_ injection (Figure 5e), consistent with biodistribution study findings. In the free ^99m^Tc control group, significant radiation uptake was observed in the thyroid (white arrows), kidney (red arrows), and bladder (yellow arrows), but no uptake was observed in tumors at 1 and 3 h post-injection of free ^99m^Tc (Figure A2). Furthermore, scintigraphy results revealed considerable radioactive uptake in kidneys and bladders of all mice injected with ^99m^Tc-(HE)_3_Z_HER2:V2_, consistent with biodistribution study observations. Immunohistochemical staining confirmed that SKOV3 and MDA-MB-361 tumor tissues strongly expressed HER2, while AT-3 tumors were HER2-negative, consistent with experimental expectations.

## 4 Discussion

The oncogenic mechanisms of HER2, also known as receptor tyrosine protein kinase erbB-2, include promoting tumor cell proliferation, tumor angiogenesis, increasing tumor cell invasiveness, and inhibiting tumor cell apoptosis [16]. HER2 is a highly expressed tumor marker in various malignant tumors, including epithelial ovarian cancer and breast cancer, and its overexpression is significantly associated with poor patient prognosis [17]. Currently, a variety of radionuclide-labeled HER2 affibodies or antibodies have been utilized in preclinical and clinical research involving HER2-positive SKOV3 ovarian and breast cancers [18,19].

The initial focus of research and development in molecular probes targeting HER2 receptor imaging was on radionuclide-labeled monoclonal antibodies. However, these antibodies, due to their complex structure and large molecular mass (approximately 150 kDa), exhibit poor thermal stability and involve a complicated preparation process [20]. In contrast, radionuclide-labeled HER2 affibody molecular probes offer several advantages, including smaller relative molecular weight, simple structure, high specificity and affinity, strong tissue penetration, and rapid concentration at the target site, enabling the acquisition of high-contrast images shortly after injection [10,19]. In an early study, an indirect labeling method involved attaching the chelating agent maGGG (mercaptoacetyl-glycyl-glycyl-glycyl) to the N-terminal of the parent Z_HER2:342_ affibody, followed by ^99m^Tc labeling. The resulting molecular probe, ^99m^Tc-MAG3-Z_HER2:342_, was effective for imaging HER2-positive SKOV3 ovarian cancer. However, a notable drawback was its high non-specific uptake in the liver and gastrointestinal tract, potentially affecting the detection of abdominal lesions [21]. To address this issue, the team refined the study design and compared ^99m^Tc labeling using various chelators: maGSG, maGEG (mercaptoacetyl-glycylseryl-glutamyl-glycylseryl), maEEE (mercaptoacetyl-glutamyl-glutamyl-glutamyl), maESE (mercaptoacetyl-glutamyl-seryl-glutamyl), maEES (mercaptoacetyl-glutamyl-glutamyl-seryl), maSEE (mercaptoacetyl-seryl-glutamyl-glutamyl), maSKS (mercaptoacetyl-seryl-lysyl-seryl), and maKKK (mercaptoacetyl-lysyl-lysyl-lysyl). The results indicated that the molecular probe ^99m^Tc-maESE-Z_HER2:342_, utilizing maESE as a chelator, minimized radiation uptake in non-target organs such as the liver, kidney, and gastrointestinal tract, thereby enhancing the quality of abdominal images [22–24]. Previously, most studies employed the “incubate in boiling water” method, complicating the process of labeling ^99m^Tcon affibody molecules [22,25,26]. Yang et al. subsequently used a direct labeling method for affibody Z_HER2:V2_ with ^99m^Tc, demonstrating specific and efficient targeting of tumor imaging in HER2-overexpressing contexts [1]. Nonetheless, high liver radiation uptake remained a drawback. In our study, we labeled ^99m^Tc after incorporating the HEHEHE sequence at the amino terminus of affibody Z_HER2:V2_. This modification enabled the molecular probe to target HER2-positive SKOV3 ovarian cancer and MDA-MB-361 breast cancer xenografts for imaging. Importantly, the labeling process was simplified, and liver radioactive uptake decreased rapidly, addressing previous study limitations.

In the present study, HER2-positive SKOV3 ovarian cancer and MDA-MB-361 breast cancer cell lines were implanted in the right armpit of Balb/C nude mice. Subsequently, freshly prepared ^99m^Tc-(HE)_3_Z_HER2:V2_ was administered via the tail vein for SPECT imaging. Results indicated notable radioactive concentration in the tumors at 1 and 3 h post-injection of ^99m^Tc-(HE)_3_Z_HER2:V2_, confirming its HER2 receptor-targeting capability. Importantly, this study demonstrated that patients can undergo imaging shortly (within 1 h) after drug injection, with an optimal imaging time window of 1–3 h. One hour post-injection of ^99m^Tc-(HE)_3_Z_HER2:V2_, significant radioactive accumulation was observed in the bladder and kidneys, which decreased by the 3 h imaging time point. This suggests predominant renal excretion of the molecular probe. Patients were advised to void as much as possible before imaging to enhance lesion detection near the kidneys and bladder, while reducing residual body radioactivity. Biological activity measurements revealed higher affinity of ^99m^Tc-(HE)_3_Z_HER2:V2_ for the MDA-MB-361 cell line compared to SKOV3. However, during imaging, SKOV3 tumors exhibited higher radioactive uptake than MDA-MB-361 tumors, possibly due to the larger volume of SKOV3 tumors at the time of imaging. Additionally, biodistribution experimental data of ^99m^Tc-(HE)_3_Z_HER2:V2_ in SKOV3 xenografts and imaging outcomes in both SKOV3 and MDA-MB-361 xenografts revealed significant radioactive accumulation in the bladder, kidneys, and tumors. Early post-injection, the liver also exhibited notable radioactive uptake, which decreased rapidly within 1 h. Conversely, non-target tissues such as spleen, heart, lungs, muscles, bones, thyroid glands, and brain showed minimal radioactive uptake, contributing to a high target-to-background ratio. Notably, tumor radioactive uptake continued to rise at 4 h post-injection in contrast to gradual decline in other organ tissues including blood. This prolonged tumor retention may be related to the strong affinity of ^99m^Tc-(HE)_3_Z_HER2:V2_’s to tumors. Moreover, high radiation retention in the kidneys also led to further uptake of tumors. In contrast, non-specific binding in other organs resulted in diminishing radioactive levels over time.

The study had several limitations, including the use of SPECT, which is a human imaging system, resulting in suboptimal image quality. Additionally, the higher tracer uptake in the kidneys could potentially increase renal radiation exposure. In clinical settings, encouraging patients to increase fluid intake can enhance tracer excretion.

In conclusion, the HER2 affibody (HE)_3_Z_HER2:V2_, prepared through recombinant gene expression, demonstrates efficient labeling with ^99m^Tc, achieving a yield exceeding 95% at room temperature. The labeling process is straightforward, yielding stable physicochemical properties. ^99m^Tc-(HE)_3_Z_HER2:V2_ effectively targets HER2-positive SKOV3 and MDA-MB-361 xenografts in imaging studies, allowing for imaging shortly after injection with an optimal window of 1–3 h. This results in specific radioactive uptake in tumors, highlighting the potential of this molecular probe for HER2-positive tumor targeting.
